# Role of local and distant functional connectivity density in the development of minimal hepatic encephalopathy

**DOI:** 10.1038/srep13720

**Published:** 2015-09-02

**Authors:** Rongfeng Qi, Long Jiang Zhang, Hui Juan Chen, Jianhui Zhong, Song Luo, Jun Ke, Qiang Xu, Xiang Kong, Chang Liu, Guang Ming Lu

**Affiliations:** 1 Department of Medical Imaging, Jinling Hospital, Clinical School of Medical College, Nanjing University, Nanjing, Jiangsu, 210002, China; 2 Center for Brain Imaging Science and Technology, Zhejiang University, Hangzhou, 310027, China; 3 Department of Gastroenterology, Jinling Hospital, Clinical School of Medical College, Nanjing University, Nanjing, Jiangsu, 210002, China

## Abstract

The progression of functional connectivity (FC) patterns from non-hepatic encephalopathy (non-HE) to minimal HE (MHE) is not well known. This resting-state functional magnetic resonance imaging (rs-fMRI) study investigated the evolution of intrinsic FC patterns from non-HE to MHE. A total of 103 cirrhotic patients (MHE, n = 34 and non-HE, n = 69) and 103 healthy controls underwent rs-fMRI scanning. Maps of distant and local FC density (*d*FCD and *l*FCD, respectively) were compared among MHE, non-HE, and healthy control groups. Decreased *l*FCD in anterior cingulate cortex, pre- and postcentral gyri, cuneus, lingual gyrus, and putamen was observed in both MHE and non-HE patients relative to controls. There was no difference in *l*FCD between MHE and non-HE groups. The latter showed decreased *d*FCD in inferior parietal lobule, cuneus, and medial frontal cortex relative to controls; however, MHE patients showed decreased *d*FCD in frontal and parietal cortices as well as increased *d*FCD in thalamus and caudate head relative to control and non-HE groups. Abnormal FCD values in some regions correlated with MHE patients’ neuropsychological performance. In conclusion, *l*FCD and *d*FCD were perturbed in MHE. Impaired *d*FCD in regions within the cortico-striato-thalamic circuit may be more closely associated with the development of MHE.

Minimal hepatic encephalopathy (MHE), a common complication of liver cirrhosis, is characterized by the presence of cognitive alterations that are undiagnosed by routine clinical examination and identified solely through neurological or psychometric tests^1^. MHE occurs with high prevalence (30% to 84%) in cirrhotic patients^2^,^3^. MHE patients have markedly reduced health-related quality of life, impaired ability to work, increased risk of falling and traffic accidents, as well as poor survival^4^. Therefore, early diagnosis of MHE is important for timely intervention and improved prognosis for these patients^5^. However, the neuropathological mechanisms of MHE remain unclear.

Abnormalities in the cortico-striato-thalamic loop have been linked to MHE^6^,^7^,^8^, as evidenced by convergent neuroimaging studies documenting the redistribution of glucose metabolism, ammonia, and cerebral blood flow from various cortical regions to the thalamus and basal ganglia^6^,^8^,^9^, as well as disturbed functional connectivity between regions within this circuit in HE patients^10^. A recent report investigating changes in whole-brain functional connectivity found that it was weaker within the cortico-striato-thalamic pathway in cirrhotic patients with MHE than in healthy controls^10^. However, it is unclear how this circuit is linked to the progression of non-HE to MHE. Only a few studies have directly compared functional changes in the brain between MHE and non-HE patients^11^,^12^,^13^. A gradual reduction in functional connectivity from non-HE to MHE was established in a brain default mode network (DMN)^11^,^12^. Another study compared spontaneous brain activity between non-HE and MHE subjects based on regional homogeneity (ReHo) and found that a lower ReHo value in the supplementary motor area and cuneus was associated with the development of MHE^13^. However, these approaches have examined connectivity within a single network (e.g., the DMN) or a limited anatomical distance (e.g., the ReHo), and thereby overlooked alterations in the whole brain network. In the present study, we used a novel functional connectivity density (FCD)^14^ approach that measures the strength of intrinsic connectivities between one voxel and others within the whole brain in order to investigate the progression from non-HE to MHE. Additionally, a recently developed method was used to explore differentiation between the distant FCD (*d*FCD) and local FCD (*l*FCD) of all brain areas according to their anatomical distance^15^. In summary, the FCD approach investigates the extensive whole-brain, distant, or local connectivity throughout the brain in an unbiased manner^16^.

We hypothesized that in cirrhotic patients, the reorganization of FCD within the cortico-striato-thalamic pathway contributes to the development of MHE.

## Results

### Clinical data and neuropsychological tests

No differences in gender, age, or education level were found between patients and control subjects (P > 0.05). However, as expected, compared to healthy controls, cirrhotic patients had worse neuropsychological performance (P < 0.05); that is, a longer time to complete the NCT-A, and lower DST scores (Table 1).

Functional MRI data from one MHE patient and one control were excluded due to marked head movement; therefore, 33 MHE patients, 69 non-HE patients, and 102 control subjects were included in the final analysis.

### FCD results

**Figure 1:**
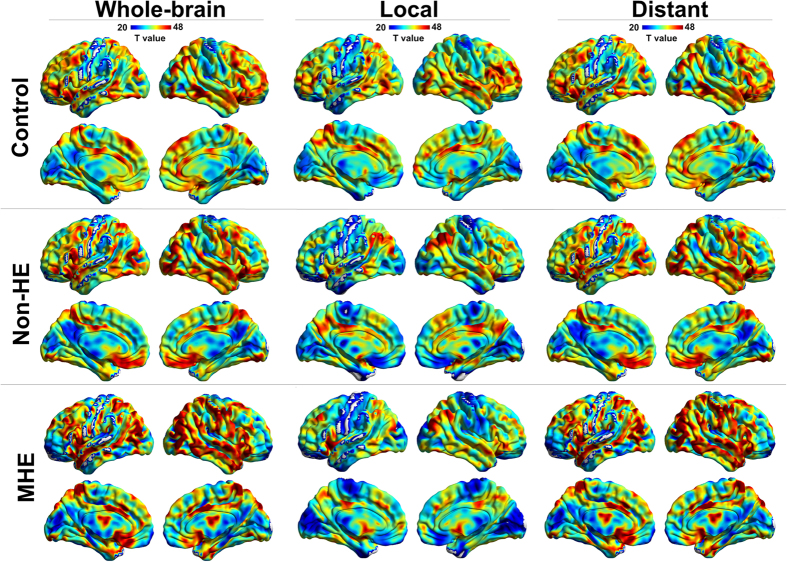
FCD maps in control, non-HE, and MHE groups. Total FCD, *l*FCD, and *d*FCD maps in each group are shown in the first, middle, and last columns, respectively. Within each group, DMN regions (including mPFC, PCC/PCu, and lateral temporal and parietal cortices), and dorsolateral prefrontal, anterior cingulate, and visual cortices show high FCD values. MHE = minimal hepatic encephalopathy; non-HE = non-hepatic encephalopathy; *l*FCD = local functional connectivity density; *d*FCD = distant functional connectivity density; mPFC = medial prefrontal cortex; PCC/PCu = posterior cingulate cortex/precuneus.

FCD patterns in control subjects were bilateral and maximal in the DMN regions including medial frontal and parietal cortices (e.g., medial prefrontal cortex and posterior cingulate cortex/precuneus) and lateral parietal and temporal cortices (e.g., inferior parietal lobe and superior temporal gyrus), regions that were previously identified as cortical hubs^16^. The patterns were also bilateral in the dorsolateral prefrontal, anterior cingulate, and visual cortices. FCD patterns in the non-HE and MHE groups were similar to that of the control group (Fig. 1). These results were visualized with BrainNet Viewer^17^ (http://www.nitrc.org/projects/bnv/).

**Figure 2:**
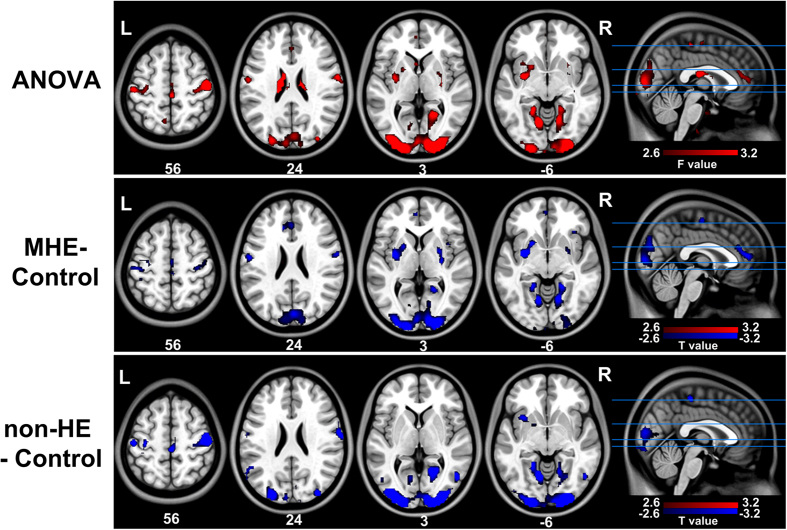
Between-groups differences in local FCD maps. Differences in *l*FCD maps are observed in pre- and postcentral gyri, the cuneus, putamen, and lingual gyrus. MHE and non-HE patients have decreased *l*FCD in these regions relative to control subjects, although there are no differences between patient groups. *l*FCD = local functional connectivity density; MHE = minimal hepatic encephalopathy; non-HE = non-hepatic encephalopathy.

**Figure 3:**
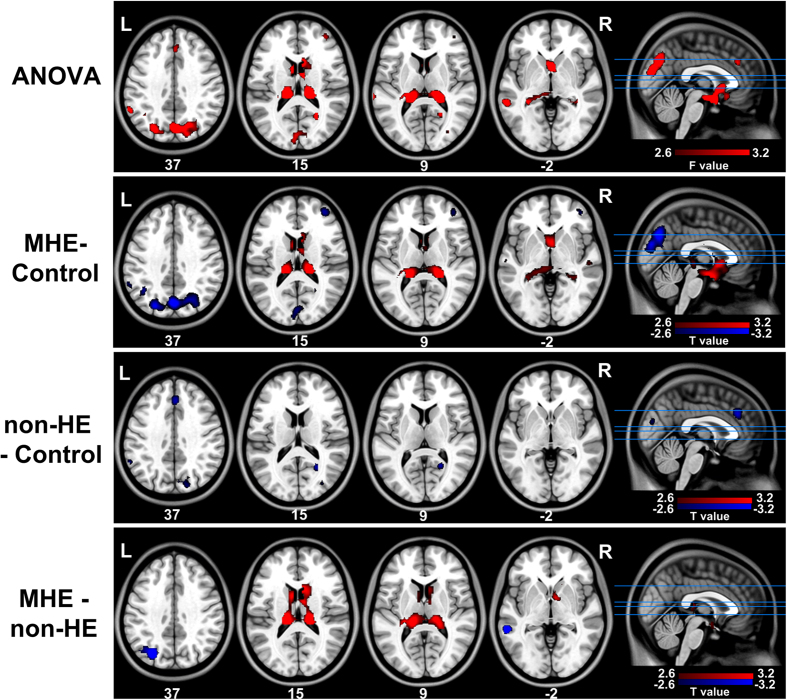
Between-groups differences in *d*FCD maps. Differences across groups are detected in the IPL, cuneus, precuneus, MTG, right middle frontal, thalamus, and caudate head regions by ANOVA. Decreased *d*FCD in the left IPL, right cuneus, and medial frontal cortex is observed in non-HE patients relative to controls. MHE patients show decreased *d*FCD in frontal and parietal cortices but increased *d*FCD in bilateral thalami and the caudate head as compared to non-HE and control groups. *d*FCD = distant functional connectivity density; ANOVA = analysis of variance; MHE = minimal hepatic encephalopathy; non-HE = non-hepatic encephalopathy; IPL = inferior parietal lobule; MTG = middle temporal gyrus.

ANOVA results revealed differences in the FCD maps among the three groups. For *l*FCD (Fig. 2, Table 2, Supplemental Tables I-II), these were mainly located in pre- and postcentral gyri, cuneus, putamen, and lingual gyrus; both MHE and non-HE groups showed decreased *l*FCD in these regions as compared to the control group but were similar to each other. For *d*FCD (Fig. 3, Table 3, and Supplemental Tables III–V), differences were detected in the inferior parietal lobule (IPL), cuneus, precuneus, middle temporal gyrus, right middle frontal cortex, thalamus, and caudate head. Compared to healthy subjects, the non-HE group showed decreased *d*FCD in the left IPL, right cuneus, and medial frontal cortex, but there was no increase in *d*FCD. In addition, when compared to the non-HE and control groups, *d*FCD was decreased in several frontal and parietal cortices and increased in bilateral thalami and the caudate head in MHE group. Since total and *d*FCD maps were highly correlated, results from the total maps are presented only as supplementary materials (Supplemental Fig. 1 and Supplemental Tables VI–IX).

### Correlations results

**Figure 4:**
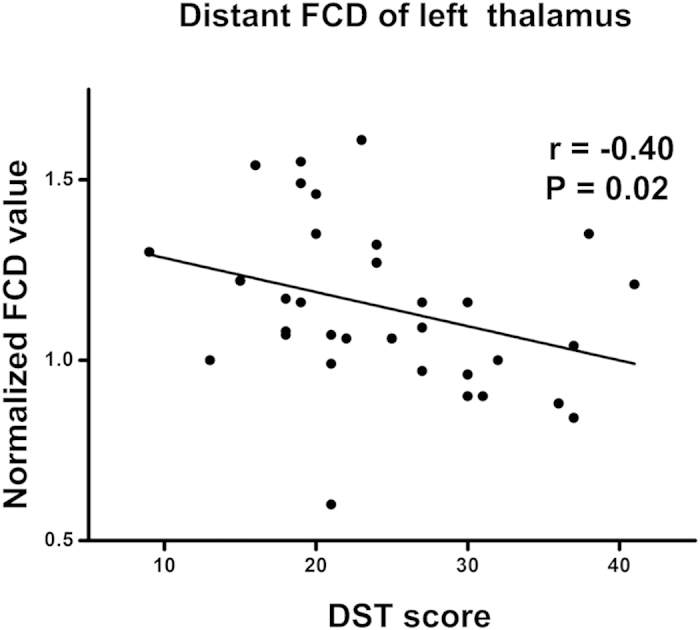
Correlation between neuropsychological performance and abnormal FCD. There is a slight negative correlation in MHE patients between DST scores of MHE patients and *d*FCD in left thalamus (uncorrected P < 0.05), but after multiple corrections this is not statistically significant. *d*FCD = distant functional connectivity density; DST = digit symbol test; MHE = minimal hepatic encephalopathy.

A slight negative correlations were found between MHE patients’ DST scores and *d*FCD in left thalamus (r = −0.40, P = 0.02) (Fig. 4), as well as total FCD in left thalamus (r = −0.38, P = 0.03) (Supplementary Fig. 2), but after multiple correction these were not statistically significant.

## Discussion

The current study investigated network alterations in cirrhotic patients to characterize the progression from non-HE to MHE. We found that *l*FCD decreased in many cortical regions in non-HE patients, while no difference was detected between non-HE and HE patients; moreover, *d*FCD decreased in several cortical regions in non-HE patients but was significantly altered by the appearance of MHE (mainly within the cortico-striato-thalamic loop). These results indicate that the *l*FCD and *d*FCD reflect different aspects of the progression from non-HE to MHE: *l*FCD may be a sensitive biomarker of the impact of cirrhosis on the brain, while *d*FCD is more specific to the appearance of MHE.

### Comparison of FCD with other functional connectivity strategies

Over the past two decades, various studies have investigated functional connectivity between spatially segregated brain regions in healthy subjects as well as in patients with different psychiatric and neurological disorders^18^,^19^. Specific analytical strategies examine different aspects of brain function. For example, a seed-based correlation analysis measures correlations between time series to identify brain areas that are connected to seed regions, relying heavily on prior selection of particular seed regions^20^. Independent component analysis, a data-driven technique, separates a set of spatially independent maps/components from mixed blood oxygen level-dependent signals, focusing on integrated coherence within each independent component (network)^21^. Compared to these methods, the FCD algorithm has the advantage of identifying hubs across the whole brain in an unbiased manner^14^, as shown here and in several other studies^16^,^22^.

### Abnormal *l*FCD in cirrhotic patients

In this study, the MHE and non-HE groups showed decreased *l*FCD in the cuneus, lingual gyrus, pre- and postcentral gyri, and putamen. The cuneus is considered to be critical for visual processing and inhibitory control^23^; the lingual gyrus works with the cuneus in visuospatial ability, somatosensory stimulation, and perception of sensory stimuli^24^; pre- and postcentral gyri are important components of motor and sensory areas; and the putamen is linked to motor performance, especially the automatic execution of previously learned movements^25^. There have been several neuroimaging studies of patients with cirrhosis showing decreased functional connectivity, activity or cerebral blood flow in gray matter areas, including the above-mentioned regions^6^,^8^,^26^. Cirrhosis affects patients’ cognitive function in the domains of visual processing and attention, as well as psychomotor, vigilance, and integrative functions^27^,^28^,^29^. The results presented here are consistent with those of previous studies^6^,^8^,^26^ and provide insight into the effects of cirrhosis on the brain.

It is worth noting that *l*FCD did not differ between MHE and non-HE groups, suggesting that abnormal *l*FCD may be a sensitive but not specific biomarker for MHE. However, a previous fMRI study^30^ reported decreased ReHo—an index for regional signal similarity in a time series of a given voxel and its nearest 26 neighboring voxels—in MHE relative to non-HE patients, and proposed that the cuneus may serve as a specific marker for MHE. Differences in sample size and computational techniques may account for these discrepancies^31^.

### Abnormal *d*FCD in cirrhotic patients

The *d*FCD was decreased in frontal and parietal cortices (e.g., cuneus and medial frontal cortex) in both non-HE and MHE patients. However, only MHE patients showed higher *d*FCD in the thalamus and caudate. These findings are consistent with previous position emission tomography studies showing that in MHE patients, glucose metabolism and ammonia as well as cerebral blood flow were redistributed from various cortical regions to the thalamus and basal ganglia^6^,^8^,^9^; it is also partially supported by the observed correlations between regional FCD and neuropsychological performance, where higher *d*FCD in the thalamus was slightly correlated with lower DST performance in MHE patients. The thalamus is a critical component of the cortical-basal ganglia-thalamic brain circuit, which may serve as a filter for sensory inputs from the cortex^32^. Besides increased perfusion in the thalamus, previous structural studies have also reported higher thalamic volumes in patients with a history of overt HE^33^ and MHE^34^, suggesting that higher numbers of thalamic neurons play a compensatory role in these patients. The caudate plays an important role in evaluating the consequence of actions and in the transmission of sensory information^35^. Brain imaging studies in HE patients have implicated the cortico-striato-thalamic pathway in the pathophysiology of HE^6^,^7^,^8^. In addition, some studies have shown an inverse correlation between neuropsychological test performance and spontaneous brain activity in the caudate^13^ and cerebral blood flow in basal ganglia^8^. Based on these findings, we speculate that the higher *d*FCD in the thalamus and caudate may be an indicator for the appearance of MHE in cirrhotic patients that results from a compensatory response to reduced functional connectivity in the cortex.

This study had some limitations. First, caution must be applied when drawing inferences from FCD abnormalities in this study since the cirrhosis occurs for a multitude of reasons. Longitudinal studies are needed to disentangle the relationship between MHE and brain functional abnormalities, and to address whether the observed results are altered by MHE treatment. Second, only two neuropsychological tests were used; future studies will include a broader spectrum of tests to evaluate the various cognitive domains of cirrhotic patients. Third, since the parameters in the FCD analysis were inconsistent, only default parameters in the DPARSF software^36^ and from a previous fMRI study^37^ were used. In addition, there is also controversy about the global signal regression in rs-fMRI data preprocessing^38^,^39^,^40^. The effect of using different FCD and preprocessing parameters on the results will be examined in later studies.

In conclusion, MHE patients showed perturbations in *l*FCD and *d*FCD in various regions within the cortico-striato-thalamic circuit; the latter could may be more closely associated with the development of MHE.

## Materials and Methods

### Subjects

This prospective study was approved by the Jinling hospital Medical Research Ethics Committee, and all experiments were performed in accordance with relevant guidelines and regulations. Written, informed consent was obtained from all subjects prior to the study. A total of 103 cirrhotic patients (mean age, 48.0 years; 81 men, 22 women; all right-handed) were recruited from June 2009 to January 2014. Inclusion criteria were as follows: (a) with clinically proven cirrhosis; (b) age older than 18 years; (c) without clinical signs of HE; (d) without magnetic resonance imaging (MRI) contraindications. Exclusion criteria were as follows: (a) any obvious brain lesion such as tumor or stroke; (b) history of drug abuse; (c) head motion of more than 1.0 mm in translation or a greater than 1.0° rotation during MRI.

The diagnosis of MHE was made according to a final report of the working group of the 11^th^ World Congress of Gastroenterology in Vienna in 1998^41^ and the 2014 guidelines of the American Association for the Study of Liver Diseases and the European Association for the Study of the Liver^42^. Each subject was administered two standard neuropsychological tests—that is, the number connection type-A (NCT-A) and digit symbol test (DST)—prior to undergoing the MRI scan. Cirrhosis patients were considered as MHE if their scores on at least one neuropsychological test were abnormal (>two standard deviations from the mean value of age-matched controls)^30^,^43^. Based on this criterion, 34 of 103 cirrhosis patients (33.0%) had MHE.

Patients completed laboratory tests 1 week before the MRI scan. A protein metabolism test, prothrombin time, and venous blood ammonia level were measured to assess the severity of liver disease. The grade of hepatic function was evaluated according to the Child-Pugh score^44^; 58/103patients were grade A, 42/103 were grade B, and 3/103were grade C.

In addition, 103 healthy controls (mean age: 47.4 years; 72 men, 31 women; all right-handed), frequency matched in age and gender, were recruited from the community by advertisement. Control subjects had no liver or other systemic diseases. Other exclusion criteria were the same as those applied to patients. Control subjects underwent neuropsychological testing prior to the MRI scan but no laboratory tests were performed.

### MRI data acquisition

Subjects were scanned using a 3 Tesla MR scanner (TIM Trio; Siemens Medical Solutions, Erlangen, Germany). A foam pad was used to minimize head motion. Resting-state functional images were obtained using a gradient-recalled echo-planar imaging sequence (250 volumes; repetition time/echo time = 2000 ms/30 ms; field of view = 240 × 240 mm; flip angle = 90°; section thickness = 4 mm; matrix = 64 × 64; 30 axial slices covering the whole brain).

### Data preprocessing

The preprocessing of functional MR images was performed using DPARSF software^36^. The first 10 volumes were discarded, and the remaining 240 images were corrected for temporal differences and head motion. Data from one MHE patient and one healthy control was discarded because of excessive head motion. Therefore, 33 MHE patients, 69 non-HE patients, and 102 healthy control subjects were included in the analysis. There were no differences in terms of translation, rotation, or motion spikes numbers^45^ among three groups (P > 0.05 for each parameter with one-way analysis of variance). Functional images were subsequently normalized to a standard stereotaxic space (3 × 3 × 3 mm^3^ from the standard Montreal Neurological Institute space). Linear detrending and temporal bandpass filtering (0.01–0.08 Hz) were performed to reduce the effects of low-frequency drift and high-frequency physiological noise using REST1.8 software (http://resting-fmri.sourceforge.net). Before functional connectivity analysis, several sources of spurious variance—including six head motion parameters obtained by rigid body head motion correction as well as average signals from cerebrospinal fluid, white matter, and whole brain—were removed using a linear regression process^46^.

### FCD analysis

To measure the *d*FCD and *l*FCD of each voxel throughout the brain, a voxel-wise whole-brain correlation analysis with a correlation threshold of r = 0.25 was performed for each subject^16^,^37^. The FCD of a voxel was then calculated as the sum of connectivity (r values) between a given voxel and others, in a manner analogous to weighted density centrality in the graph theory of the brain^22^,^47^. Voxels with higher FCD values indicated a central role in information transfer through the brain. For standardization purposes, the FCD of each voxel was divided by the global mean connectivity density value. In addition, a neighborhood strategy was chosen to define *l*FCD and *d*FCD^37^. For *l*FCD, voxels in a 3 mm-radius sphere (comprising seven voxels) surrounding the seed voxel were included^37^. For *d*FCD, all voxels outside a 25-mm-radius sphere were included^37^. The gap between *l*FCD and *d*FCD distances excluded any possible overlap between indices.

### Statistical analysis

SPSS 16.0 (SPSS Inc., Chicago, IL, USA) was used to analyze demographic and clinical data. SPM8 was used to smooth FCD maps with an 8-mm kernel and analyze the smoothed connectivity maps at the group level. Within each group, a random effects one-sample t test was performed on individual *l*FCD and *d*FCD maps. Significant thresholds were set at a corrected P value < 0.05 using the AlphaSim program (http://afni.nimh.nih.gov/pub/dist/doc/manual/AlphaSim.pdf).

To examine differences among the three groups, one-way analysis of variance (ANOVA) was performed to determine differences of whole brain (total), local, and distant FCD maps, followed by post-hoc t tests to examine between-groups differences within significant regions detected by ANOVA, while eliminating the effects of age, gender, and education level by regression. Statistical thresholds were set at P < 0.05 and corrected using the AlphaSim program.

To investigate the association between neuropsychological performance, clinical indices, and FCD in patients, mean FCD values of regions that differed significantly among the three groups (ANOVA results) were extracted and correlated with NCT-A and DST scores, serum albumin, prothrombin time, and venous blood ammonia levels of non-HE and MHE patients, separately, using Spearman correlation analysis, meanwhile eliminating the effects of age, sex, and education by regression. Correlations were significant for P values < 0.05 and were corrected for multiple comparisons using the Bonferroni correction for the number of regions where altered FCD was detected among three groups (cutoff P values of 0.05/13, 0.05/9, and 0.05/13 were performed for total FCD, lFCD, and dFCD respectively, corresponding to 13, 9, and 13 regions showing differences in these three measurements).

## Additional Information

**How to cite this article**: Qi, R. *et al.* Role of local and distant functional connectivity density in the development of minimal hepatic encephalopathy. *Sci. Rep.* **5**, 13720; doi: 10.1038/srep13720 (2015).

## Supplementary Material

Supplementary file

## Figures and Tables

**Table 1:** Demographics and clinical data of all cirrhotic patients and healthy controls.

Protocols	HC (n = 103)	Patients (n = 103)	P value
Gender(M/F)	72/31	81/22	0.15^a^
Age (±SD), y	47.43 ± 10.09	47.99 ± 10.27	0.69^b^
Education, y	10.97 ± 3.18	10.45 ± 3.11	0.82^b^
Venous blood ammonia level (u mol/L)		53.34 ± 34.34	
Child-Pugh scale (n)			
A		58	
B		42	
C		3	
NCT-A (s)	44.06 ± 10.66	54.38 ± 20.02	<0.001^b^
DST (score)	46.83 ± 12.51	35.52 ± 11.80	<0.001^b^
MHE patients (n)		34	
Non-HE patients (n)		69	

^a^The P value for gender distribution in the two groups was obtained by Chi-square test.

^b^The P value for age and neuropsychological tests difference between the two groups was obtained by two sample t test.

Values are expressed as mean ± SD. NCT-A = number connection test type A; DST = digit symbol test; HC = healthy control; MHE = minimal hepatic encephalopathy; non-HE = non-hepatic encephalopathy.

**Table 2:** Regions showing local functional connectivity density differences among the MHE, non-HE patients, and healthy controls.

Brain regions	BA	MNI coordinates (mm)	Vol (mm^3^)	Maximal F value
		(x, y, z)		
Left ACC	32/10	0, 48, 9	61	6.83
Left pre- and postcentral gyri	3/4	−48, −12, 60	148	14.96
Right pre- and postcentral gyri	3/4	48, −21, 51	177	13.20
Left cuneus	18	−24, −89, 23	63	9.58
Right cuneus	18	15, −93, 13	94	8.56
Left putamen	7	−27, 0, 0	61	7.68
Right putamen	7	27, 0, 0	56	8.78
Left lingual gyrus	19	−12, −63, −6	79	16.41
Right lingual gyrus	19	15, −60, −6	95	10.08

MHE = minimal hepatic encephalopathy; non-HE = non hepatic encephalopathy; BA = Brodmann area; MNI = Montreal Neurological Institute; ACC = anterior cingulate cortex. P < 0.05, corrected for multiple comparisons.

**Table 3:** Regions showing distant functional connectivity density differences among the MHE, non-HE patients, and healthy controls.

Brain regions	BA	MNI coordinates (mm)	Vol (mm^3^)	Maximal F value
		(x, y, z)		
Left IPL	40	−54, −54, 45	137	10.56
Right IPL	40	54, −54, 45	112	8.65
Left cuneus	18/19	−3, −84, 21	175	8.79
Right cuneus	18/19	7, −84, 21	226	8.80
Left precuneus	7	−27, −78, 36	55	8.68
Right precuneus	7	25, −70, 36	137	9.20
Left thalamus		−18, −36, 6	88	8.25
Right thalamus		21, −33, 9	78	16.44
Left caudate head		−6, 3, 15	91	7.63
Right caudate head		6, 15, 13	57	8.32
Left MTG	21	−66, −42, −9	64	6.06
Right MFG	10	39, 51, 6	51	6.16
Medial Frontal Cortex	9	3, 33, 30	56	7.78

MHE = minimal hepatic encephalopathy; non-HE = non hepatic encephalopathy; BA = Brodmann area; MNI = Montreal Neurological Institute; IPL = inferior parietal lobule; MTG = middle temporal gyrus; MFG = middle frontal gyrus. P < 0.05, corrected for multiple comparisons.
