# Supplementary material for: Role of local and distant functional connectivity density in the development of minimal hepatic encephalopathy
Source: Sci Rep. 2015 Sep 2;5:13720. doi: 10.1038/srep13720 (PMC4556960; doi:10.1038/srep13720)
Supplement: Supplementary file [file srep13720-s1.doc]

**Role of local and distant functional connectivity density in the development of minimal hepatic encephalopathy**

Rongfeng Qi^1^, Long Jiang Zhang^1*^, Hui Juan Chen^1^, Jianhui Zhong^2^, Song Luo^1^, Jun Ke^1^, Qiang Xu^1^, Xiang Kong^1^, Chang Liu^3^, Guang Ming Lu^1*^

*1. Department of Medical Imaging, Jinling Hospital, Clinical School of Medical College, Nanjing University, Nanjing, Jiangsu, 210002, China.*

*2. Center for Brain Imaging Science and Technology, Zhejiang University, Hangzhou, 310027, China.*

*3. Department of Gastroenterology, Jinling Hospital, Clinical School of Medical College, Nanjing University, Nanjing, Jiangsu , 210002, China.*

* Correspondence to: Long Jiang Zhang, M.D., Guang Ming Lu, M.D., Department of Medical Imaging, Jinling Hospital, Clinical School of Medical College, Nanjing University, 305 Zhongshan East Road, Xuanwu District, Nanjing, Jiangsu Province 210002, China.

Tel: 86-25-80860185. Fax: 86-25-84804659.

Email: kevinzhlj@163.com; [cjr.luguangming@vip.163.com](mailto:cjr.luguangming@vip.163.com)

Competing interests: None of the authors has any conflict of interest to disclose.

**Table S-I:** Brain regions showing local functional connectivity density differences between MHE patients and healthy controls

| Brain regions | BA | MNI coordinates (mm) | Vol (mm^3^) | Maximal T value^#^ |
| --- | --- | --- | --- | --- |
|  |  | (x, y, z) |  |  |
| Left ACC | 32/10 | -3,45,9 | 61 | -3.54 |
| Left pre- and postcentral gyri | 3/4 | -57,-12,33 | 206 | -3.91 |
| Right pre- and postcentral gyri | 3/4 | 60,-3,30 | 130 | -4.56 |
| Left cuneus | 18 | -15,-93,30 | 74 | -3.59 |
| Right cuneus | 18 | 15,-96, 12 | 145 | -4.09 |
| Left putamen | 7 | -33,0,3 | 80 | -3.58 |
| Right putamen | 7 | 27,-3,3 | 66 | -3.44 |
| Left lingual gyrus | 19 | -12,-60,-3 | 93 | -3.34 |
| Right lingual gyrus | 19 | 12,-63,-3 | 76 | -4.02 |

MHE = minimal hepatic encephalopathy; BA = Brodmann area; MNI = Montreal Neurological Institute; ACC *=* anterior cingulate cortex*.* P< 0.05, corrected for multiple comparisons. ^#^Negative value represents decreases.

**Table S-II:** Brain regions showing local functional connectivity density differences between non-HE patients and healthy controls

| Brain regions | BA | MNI coordinates (mm) | Vol (mm^3^) | Maximal T value^#^ |
| --- | --- | --- | --- | --- |
|  |  | (x, y, z) |  |  |
| Left pre- and postcentral gyri | 3/4 | -48,-18,54 | 97 | -4.01 |
| Right pre- and postcentral gyri | 3/4 | 48,-12,60 | 168 | -5.18 |
| Left cuneus | 18 | -30,-99,6 | 121 | -5.69 |
| Right cuneus | 18 | 15,-96, 12 | 94 | -5.26 |
| Left putamen | 7 | -27,12,-3 | 70 | -3.39 |
| Left lingual gyrus | 19 | -12,-60,-3 | 130 | -3.91 |
| Right lingual gyrus | 19 | 18,-60,9 | 101 | -3.78 |

non-HE = non hepatic encephalopathy; BA = Brodmann area; MNI = Montreal Neurological Institute; P< 0.05, corrected for multiple comparisons. ^#^Negative value represents decreases.

**Table S-III:** Brain regions showing distant functional connectivity density differences

between MHE patients and healthy controls

| Brain regions | BA | MNI coordinates (mm)  (x, y, z) | Vol (mm^3^) | Maximal T value^#^ |
| --- | --- | --- | --- | --- |
| Left IPL | 7/40 | -51,-54,42 | 129 | -3.29 |
| Right IPL | 7/40 | 36,-63,48 | 175 | -3.83 |
| Left cuneus | 18/19 |  | 102 | -3.39 |
| Right cuneus | 18/19 | 7,-79, 21 | 105 | -3.46 |
| Left precuneus | 7 | -27,-78,36 | 77 | -3.48 |
| Right precuneus | 7 | 25,-70,40 | 57 | -3.12 |
| Left thalamus |  | -15,-36,3 | 118 | +3.81 |
| Right thalamus |  | 18,-33,9 | 117 | +5.13 |
| Left caudate head |  | -9,24,3 | 65 | +3.28 |
| Right caudate head |  | 15,24,3 | 59 | +3.06 |
| Right MFG | 10 | 42,54,-12 | 186 | -3.39 |

MHE = minimal HE; BA = Brodmann area; MNI = Montreal Neurological Institute; IPL = inferior parietal lobule; MFG = middle frontal gyrus. *P*< 0.05, corrected for multiple comparisons. ^#^Negative and positive values represent decreases and increases, respectively.

**Table S-IV:** Brain regions showing distant functional connectivity density differences between MHE and non-HE patients

| Brain regions | BA | MNI coordinates (mm) | Vol (mm^3^) | Maximal T value^#^ |
| --- | --- | --- | --- | --- |
|  |  | (x, y, z) |  |  |
| Left IPL | 7/40 | -33,-69,45 | 109 | -3.28 |
| Right IPL | 7/40 | 36,-63,48 | 76 | -3.23 |
| Left thalamus |  | -15,-24,15 | 134 | +4.54 |
| Right thalamus |  | 18,-27,15 | 108 | +4.59 |
| Left caudate head |  | -6,6,15 | 76 | +3.59 |
| Right caudate head |  | 12,12,15 | 62 | +3.58 |
| Left MTG | 21 | -57,-39,0 | 94 | -3.37 |

MHE = minimal HE; non-HE = non hepatic encephalopathy; BA = Brodmann area; MNI = Montreal Neurological Institute; IPL = inferior parietal lobule; MTG = middle temporal gyrus. P< 0.05, corrected for multiple comparisons. ^#^Negative and positive values represent decreases and increases, respectively.

**Table S-V:** Brain regions showing distant functional connectivity density differences between non-HE patients and healthy controls

| Brain regions | BA | MNI coordinates (mm) | Vol (mm^3^) | Maximal T value^#^ |
| --- | --- | --- | --- | --- |
|  |  | (x, y, z) |  |  |
| Left IPL | 40 | -54,-54,51 | 62 | -4.69 |
| Right cuneus | 19 | 18,-81, 33 | 64 | -3.35 |
| Medial frontal cortex | 32 | 0,33,39 | 69 | -4.01 |

non-HE = non hepatic encephalopathy; BA = Brodmann area; MNI = Montreal Neurological Institute; IPL = inferior parietal lobule; P< 0.05, corrected for multiple comparisons. ^#^Negative value represents decreases.

**Table S-VI:** Brain regions showing total density of functional connectivity differences among the MHE, non-HE patients, and healthy controls

| Brain regions | BA | MNI coordinates (mm)  (x, y, z) | Vol (mm^3^) | Maximal F value |
| --- | --- | --- | --- | --- |
| Left IPL | 40 | -51,-54,45 | 139 | 11.24 |
| Right IPL | 40 | 54,-54,45 | 110 | 8.33 |
| Left cuneus | 18/19 | -3,-84,21 | 190 | 8.79 |
| Right cuneus | 18/19 | 7,-79, 21 | 238 | 8.80 |
| Left precuneus | 7 | -27,-78,36 | 55 | 8.68 |
| Right precuneus | 7 | 25,-70,40 | 136 | 9.20 |
| Left thalamus |  | -18,-36,6 | 88 | 10.08 |
| Right thalamus |  | 21,-33,9 | 80 | 9.44 |
| Left caudate head |  | -6,3,16 | 93 | 7.18 |
| Right caudate head |  | 6,15,13 | 52 | 10.19 |
| Left MTG | 21 | -60,-39,0 | 82 | 8.80 |
| Right MFC | 10 | 42,51,3 | 56 | 7.80 |
| Medial frontal cortex | 58 | 3,33,30 | 58 | 8.16 |

MHE = minimal hepatic encephalopathy; non-HE = non hepatic encephalopathy; BA = Brodmann area; MNI = Montreal Neurological Institute; IPL = inferior parietal lobule; MTG = middle temporal gyrus; MFG = middle frontal gyrus. P< 0.05, corrected for multiple comparisons.

| Brain regions | BA | MNI coordinates (mm) | Vol (mm^3^) | ^#^Maximal T value |
| --- | --- | --- | --- | --- |
|  |  | (x, y, z) |  |  |
| Left IPL | 7/40 | -51,-54,45 | 127 | -3.73 |
| Right IPL | 7/40 | 36,-63,48 | 165 | -3.52 |
| Left cuneus | 18/19 |  | 110 | -3.46 |
| Right cuneus | 18/19 | 7,-79, 21 | 95 | -3.48 |
| Left precuneus | 7 | -27,-78,36 | 58 | -3.39 |
| Right precuneus | 7 | 25,-70,40 | 78 | -3.26 |
| Left thalamus |  | -15,-24,9 | 117 | +3.67 |
| Right thalamus |  | 15,-24,9 | 118 | +3.80 |
| Left caudate head |  | -8,11,3 | 78 | +3.04 |
| Right caudate head |  | 15,24,3 | 58 | +2.82 |
| Right MFC | 10 | 42,48,3 | 183 | -3.82 |

**Table S-VII:** Brain regions showing total density of functional connectivity differences between MHE patients and healthy controls

MHE = minimal hepatic encephalopathy; BA = Brodmann area; MNI = Montreal Neurological Institute; IPL = inferior parietal lobule; MFG = middle frontal gyrus. P< 0.05, corrected for multiple comparisons. ^#^Negative and positive values represent decreases and increases, respectively.

**Table S-VIII: Brain regions showing total density of functional connectivity differences between MHE and non-HE patients**

| Brain regions | BA | MNI coordinates (mm) | Vol (mm^3^) | ^#^Maximal T value |
| --- | --- | --- | --- | --- |
|  |  | (x, y, z) |  |  |
| Left IPL | 7/40 | -51,-54,45 | 101 | -3.25 |
| Right IPL | 7/40 | 36,-63,48 | 80 | -3.10 |
| Left thalamus |  | -15,-24,9 | 98 | +3.67 |
| Right thalamus |  | 15,-24,9 | 109 | +3.80 |
| Left caudate head |  | -8,11,3 | 98 | +3.04 |
| Right caudate head |  | 15,24,3 | 52 | +2.82 |
| Left MTG | 21 | -54,-39,-3 | 96 | -3.65 |

MHE = minimal hepatic encephalopathy; non-HE =non hepatic encephalopathy; BA = Brodmann area; MNI = Montreal Neurological Institute; IPL = inferior parietal lobule; MTG = middle temporal gyrus. P< 0.05, corrected for multiple comparisons. ^#^Negative and positive values represent decreases and increases, respectively.

**Table S-IX: Brain regions showing total density of functional connectivity differences between non-HE patients and healthy controls**

| Brain regions | BA | MNI coordinates (mm) | Vol (mm^3^) | ^#^Maximal T value |
| --- | --- | --- | --- | --- |
|  |  | (x, y, z) |  |  |
| Left IPL | 40 | -60,-54,42 | 62 | 4.02 |
| Right cuneus | 19 | 15,-75, 36 | 69 | 3.42 |
| Medial frontal cortex | 32 | 3,33,30 | 66 | 3.96 |

non-HE = non hepatic encephalopathy; BA = Brodmann area; MNI = Montreal Neurological Institute; IPL = inferior parietal lobule. P< 0.05, corrected for multiple comparisons. ^#^Negative value represents decreases.


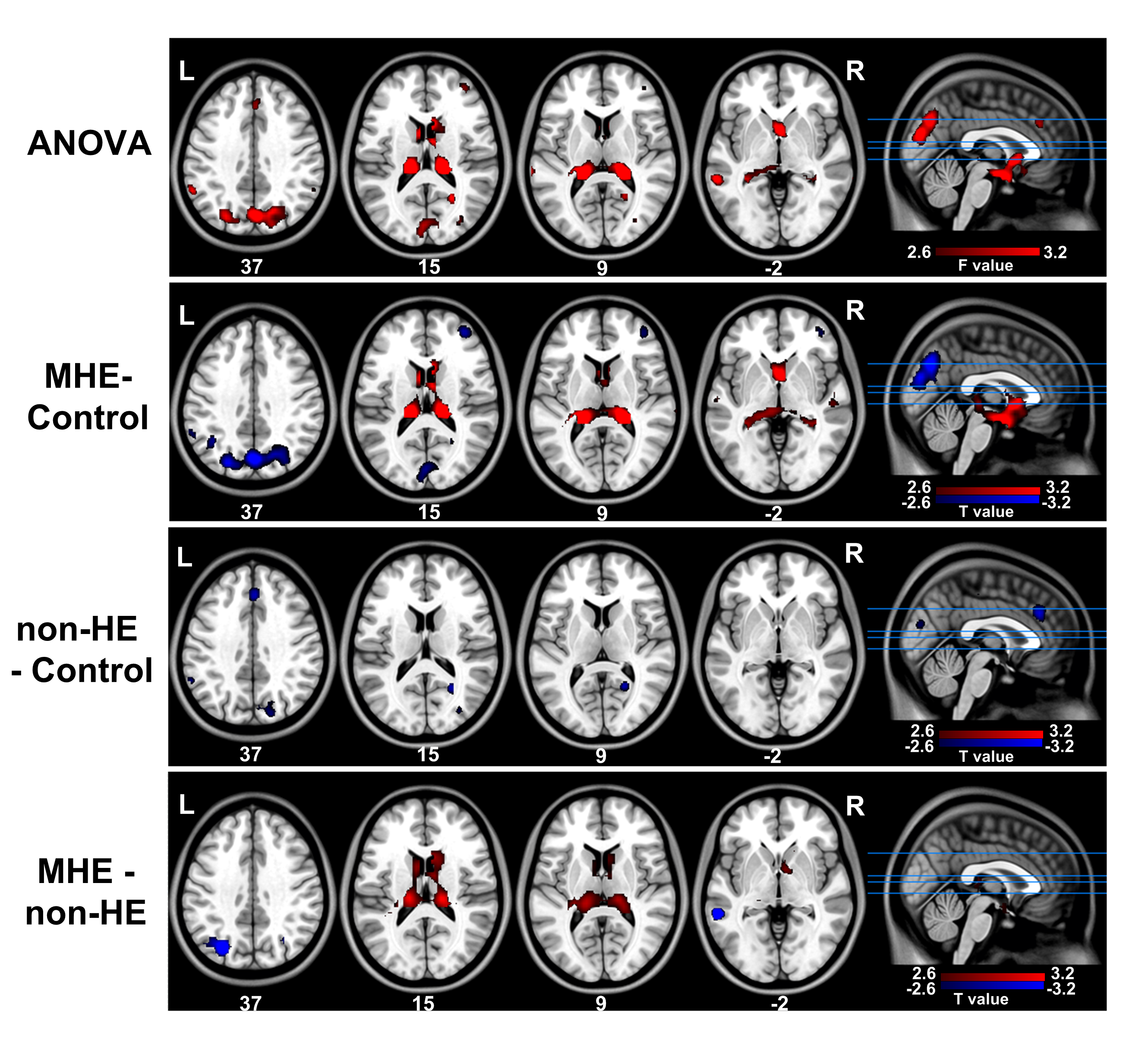


**Figure S1.**Between-groups differences of total FCD maps.

For total FCD, differences are detected in the IPL, cuneus, precuneus, MTG, right middle frontal cortex, thalamus, and caudate head. Non-HE patients show decreased total FCD in left IPL, right cuneus, and medial frontal cortex relative to healthy subjects. MHE patients show decreased total FCD in several frontal and parietal cortices and increased total FCD in bilateral thalami and the caudate head compared with non-HE patients and healthy controls.

FCD = functional connectivity density; IPL = inferior parietal lobule; MTG = middle temporal gyrus; non-HE = non-hepatic encephalopathy; MHE = minimal hepatic encephalopathy.


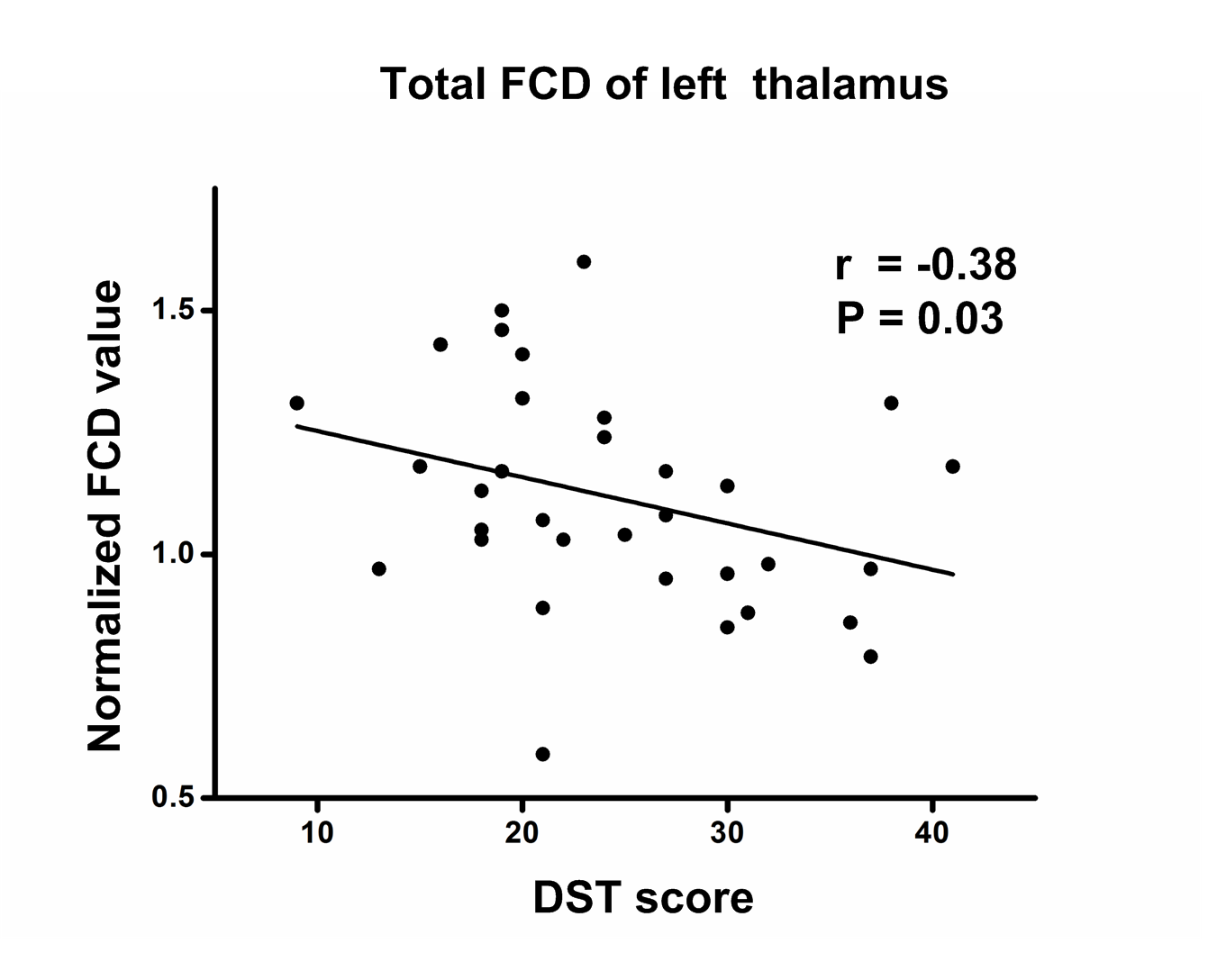


**Figure S2.**Correlation between neuropsychological performance and abnormal total FCD.

In MHE patients, there is a slight positive correlation between DST scores and total FCD in the left thalamus (uncorrected P value<0.05) but after multiple corrections this is not statistically significant.

FCD = functional connectivity density; MHE = minimal hepatic encephalopathy; DST = digit symbol test.
